# Spreading of heterochromatin and karyotype differentiation in two *Tropidacris* Scudder, 1869 species (Orthoptera, Romaleidae)

**DOI:** 10.3897/CompCytogen.v9i3.5160

**Published:** 2015-07-24

**Authors:** Marília de França Rocha, Mariana Bozina Pine, Elizabeth Felipe Alves dos Santos Oliveira, Vilma Loreto, Raquel Bozini Gallo, Carlos Roberto Maximiano da Silva, Fernando Campos de Domenico, Renata da Rosa

**Affiliations:** 1 Departamento de Biologia, ICB, Universidade de Pernambuco, Recife, Pernambuco, Brazil; 2 Departamento de Biologia Geral, CCB, Universidade Estadual de Londrina (UEL), Londrina, Paraná, Brazil; 3 Departamento de Genética, CCB, Universidade Federal de Pernambuco, Recife, Pernambuco, Brazil; 4 Museu de Zoologia, Instituto de Biociência, Universidade de São Paulo, São Paulo, São Paulo, Brazil

**Keywords:** Chromosome banding, repetitive DNA, speciation, histone H3 gene, 18S rDNA

## Abstract

*Tropidacris* Scudder, 1869 is a genus widely distributed throughout the Neotropical region where speciation was probably promoted by forest reduction during the glacial and interglacial periods. There are no cytogenetic studies of *Tropidacris*, and information allowing inference or confirmation of the evolutionary events involved in speciation within the group is insufficient. In this paper, we used cytogenetic markers in two species, *Tropidacris collaris* (Stoll, 1813) and *Tropidacris cristata grandis* (Thunberg, 1824), collected in different Brazilian biomes. Both species exhibited 2n=24,XX for females and 2n=23,X0 for males. All chromosomes were acrocentric. There were some differences in the karyotype macrostructure, e.g. in the chromosome size. A wide interspecific variation in the chromosome banding (C-banding and CMA_3_/DAPI staining) indicated strong differences in the distribution of repetitive DNA sequences. Specifically, *Tropidacris cristata grandis* had a higher number of bands in relation to *Tropidacris collaris*. FISH with 18S rDNA revealed two markings coinciding with the NORs in both species. However, two analyzed samples of *Tropidacris collaris* revealed a heterozygous condition for the rDNA site of S_10 _pair. In *Tropidacris collaris*, the histone H3 genes were distributed on three chromosome pairs, whereas in *Tropidacris cristata grandis*, these genes were observed on 14 autosomes and on the X chromosome, always in terminal regions. Our results demonstrate that, although the chromosome number and morphology are conserved in the genus, *Tropidacris cristata grandis* substantially differs from *Tropidacris collaris* in terms of the distribution of repetitive sequences. The devastation and fragmentation of the Brazilian rainforest may have led to isolation between these species, and the spreading of these repetitive sequences could contribute to speciation within the genus.

## Introduction

The genus *Tropidacris* Scudder, 1869 comprises the largest grasshoppers of the order Orthoptera, reaching up to 14 centimeters in length (Diniz-Filho et al. 2010). They have a strong influence on the food chain as prey and predators, contributing to the natural balance of the populations (Nunes 1996). These animals are of substantial ecological importance, as they are forest defoliators that feed on leaves, decaying organic matter and mosses (Amèdégnato 1977). In addition, some species can be important agricultural pests that cause extreme economic losses (Poderoso et al. 2013).

*Tropidacris* is widely spread throughout the Neotropical region. Its natural habitats extensively vary from dense rainforests to very open areas with a dry climate (Eades et al. 2010, Diniz-Filho et al. 2010). In a review, Carbonell (1986) classified the genus into three species: *Tropidacris collaris* (Stoll, 1813), *Tropidacris descampsi* (Carbonell, 1986) and *Tropidacris cristata* (Linnaeus, 1758). The latter was subdivided into three subspecies: *Tropidacris cristata dux* (Drury, 1770), *Tropidacris cristata cristata* (Linnaeus, 1758) and *Tropidacris cristata grandis* (Thunberg, 1824) (Diniz-Filho et al. 2010). All these species have different geographical distribution. *Tropidacris collaris* has a wider distribution than *Tropidacris cristata*, while the species *Tropidacris descampsi* was described based on a single specimen from Colombia (Diniz-Filho et al. 2010).

Considering the geographical distribution of the above-mentioned taxa, Carbonell (1986) proposed that their common ancestor may have arisen in the Guiano-Amazon region. He also substantiated that speciation probably began by the geographic isolation of two populations which later developed into *Tropidacris collaris* and *Tropidacris cristata*. These events probably relate to episodes of forest reduction during the glacial and interglacial retreat periods, which caused the fragmentation of this environment and its further expansion. Barriers between the allopatric populations can be created by geological forces, favoring both genetic differentiation and speciation (Nosil 2008).

In different organisms, speciation is related to important chromosomal rearrangements. Translocations, inversions, duplications and deletions can lead to chromosome segregation problems, causing different degrees of sterility. Karyotype changes, such as amplification or dispersion of repetitive DNA sequences may also have an important role in this process. These structural rearrangements lead to reproductive barriers and thus to the formation of new biological species (Livingstone and Rieseberg 2004). For this reason, reproductive barriers are extremely important in terms of evolutionary process (Southcott et al. 2013). Isolated genomes can accumulate differences until formation of a complete reproductive barrier (Rieseberg 2001, Noor et al. 2001, Livingstone and Rieseberg 2004).

There are no specific cytogenetic studies of *Tropidacris*, and information allowing inference or confirmation of the evolutionary events involved in speciation in the group is insufficient. Thus, we used cytogenetic markers to analyze *Tropidacris collaris* and *Tropidacris cristata grandis*, two members of the genus collected in different biomes of northeastern and southern Brazil. We intend to propose a mechanism that explains both the chromosome evolution and reproductive isolation between these taxa.

## Methods

### Samples and collection sites

**Figure 1.**
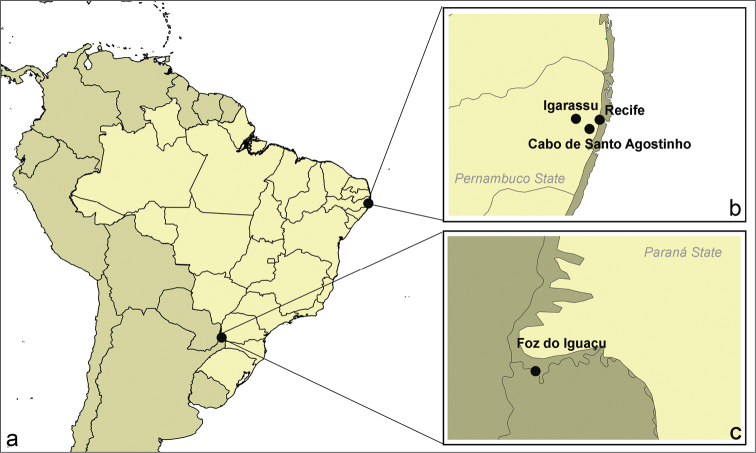
**a** map of Brazil showing collection sites in northeastern and southern Brazil **b** *Tropidacris collaris* **c** *Tropidacris cristata grandis*.

The specimens of *Tropidacris collaris* and *Tropidacris cristata grandis* were collected from two regions of Brazil (Table 1 and Fig. 1). Individuals of both species were identified and deposited in the Museu de Zoologia da Universidade de São Paulo (MZUSP).

**Table 1.** Collection sites of studied species.

Species	Number of specimens	Collection sites
*Tropidacris collaris*	4♀, 8♂ 4♀, 7♂	Refúgio Ecológico Charles Darwin, Igarassu, Pernambuco, Brazil 08°03.00'S, 35°13.00"W (DMS) Gurjaú, Cabo de Santo Agostinho, Pernambuco, Brazil 08°10'00"S, 35°05'00"W (DMS)
*Tropidacris cristata grandis*	5♀, 10♂	Iguaçu National Park, Foz do Iguaçu, Paraná, Brazil 25°37’40.67"S, 54°27’45.29"W (DMS)

### Chromosome preparation and conventional staining

The samples were anesthetized and dissected before ﬁxing their testes and gastric caeca in methanol: acetic acid 3:1. The females were injected with 0.1% colchicine 6h prior to dissection. For mitotic and meiotic analyses, air-dried chromosome preparations were made from tissues macerated in one drop of 2% lacto-acetic orcein. For banding techniques and FISH, squashed preparations with 45% acetic acid were made, and then coverslips were removed after freezing the preparations by immersion in liquid nitrogen for a few seconds.

### Chromosome banding

The distribution of heterochromatin was analyzed with Giemsa C-banding after treatments with 0.2M HCl for 15 min at 25 °C, 5% Ba(OH)2 at 60 °C in a waterbath for 1 min and 2×SSC for 30 min at 60 °C (Sumner 1972). The GC- and AT-rich bands were detected with chromomycin A_3 _(CMA_3_) and 4’-6-diamidino-2-phenylindole (DAPI), respectively (Schweizer et al. 1983). The slides were stained with chromomycin A_3_ (0.5 mg/ml in McIlvaine buffer, pH 7.0, containing 10 nM MgCl_2_) for 60 min, washed with distilled water, stained with distamycin A (0.1 mg/ml) for 30 min, again washed and finally stained with DAPI (0.5 mg/ml) for 30 min. The slides were then washed with distilled water, mounted with a 1: 1 mixture of glycerol and McIlvaine buffer, pH 7.0, and kept in the dark for at least 3 days. Silver nitrate staining of active nucleolus organizer regions (Ag-NOR) was performed according to Howell and Black (1980). Two drops of 1% aqueous gelatin solution with 0.25% formic acid and four drops of silver nitrate at 25% were placed onto the test slides, which were covered with coverslips and incubated for 7 min at 60 °C.

### Fluorescence in situ hybridization (FISH)

In addition to the karyotype studies, genomic DNA from one male of each species was extracted from the muscle tissue sample. After proteinase K (20 mg/ml) digestion for three hours at 65 °C, phenol/Tris-HCl (pH 8.0) was added, followed by centrifugation and washing with phenol/Tris-HCl (pH 8.0) and chloroform-isoamyl alcohol. After an additional centrifugation, chloroform-isoamyl alcohol was added. Then, DNA was precipitated with absolute ethanol for 12 hours at –20 °C and eluted in TE 1/10 + RNAse.

Unlabeled 18S rDNA and histone H3 gene probes were generated by polymerase chain reaction (PCR) using the following primers: 18S rDNAF 5‘-CCTGA GAAACGGCTACCACATC-3’ and 18S rDNAR 5‘-GAGTCTCGTTCGTTATCGGA-3’ (Whiting 2002); H3F 5’-ATATCCTTRGGCATRATRGTGAC-3’ and H3R 5’ATGGCTCGTACCAAGCAGACVGC-3’ (Colgan et al. 1998). The probes isolated by PCR were labeled with digoxigenin-11-dUTP by PCR. Fluorescence *in situ* hybridization was performed according to Pinkel et al. (1986) with modifications. The slides were dehydrated in an alcohol series and washed in 15% formamide/0.2×SSC, pre-treated with DNAse-free RNAse (40 µg/ml in 2×SSC) at 37 °C for 1h and with pepsin (0.005% in 10 mM HCl) at 37 °C for 30 min. Subsequently, they were fixed in 4% fresh paraformaldehyde, dehydrated in an alcohol series and air-dried. The chromosomes were then denatured in 70% formamide/2×SSC at 70 °C for 5 min. The slides were treated with 30 µl of hybridization mixture containing 100 ng of labeled probe (4 µl), 50% formamide (15 µl), 50% polyethylene glycol (6 µl), 20×SSC (3 µl), 100 ng of calf thymus DNA (1 µl) and 10% SDS (1 µl). The material was denatured at 90 °C for 10 min. Hybridization occurred overnight at 37°C in a humidified chamber. Post-hybridization washes were carried out in 2×SSC, 20% formamide in 0.1×SSC, 0.1×SSC and 4×SSC/0.2% Tween 20, all at 42 °C. The probe was detected with a solution of 5% BSA and FITC-conjugated avidin (50:0.5, v:v). The post-detection washes were performed in 4×SSC/0.2% Tween 20 at room temperature. The slides were mounted with 25 µl of a medium composed of 23 µl of DABCO solution (1,4-diazabicyclo[2.2.2] octane (2.3%), 20 mM Tris HCl, pH 8.0, (2%) and glycerol (90%), in distilled water), 1 µl of 2 µg/ml DAPI and 1µl of 50 mM MgCl_2_.

All images were acquired with a Leica DM 4500 B microscope equipped with a DFC 300FX camera and Leica IM50 4.0 software, and optimized for best contrast and brightness with iGrafx Image software.

## Results

### Tropidacris collaris

**Figure 2.**
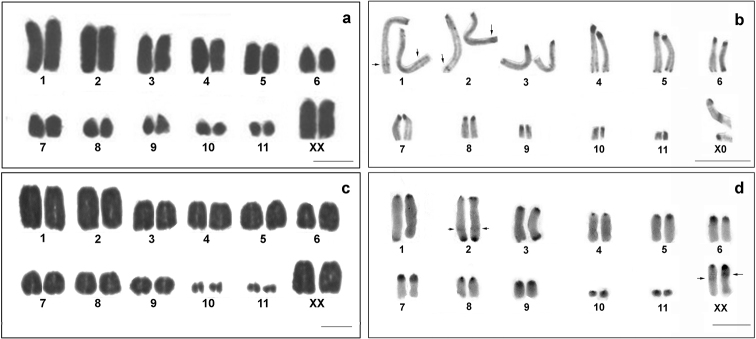
Karyotypes of the studied species. **a** female karyotype of *Tropidacris collaris*, conventional staining **b** male karyotype of *Tropidacris collaris*, C-banding **c** female karyotype of *Tropidacris cristata grandis*, conventional staining **d** male karyotype of *Tropidacris cristata grandis*, C-banding. Arrows indicate interstitial bands. Bar = 10 µm.

**Figure 3.**
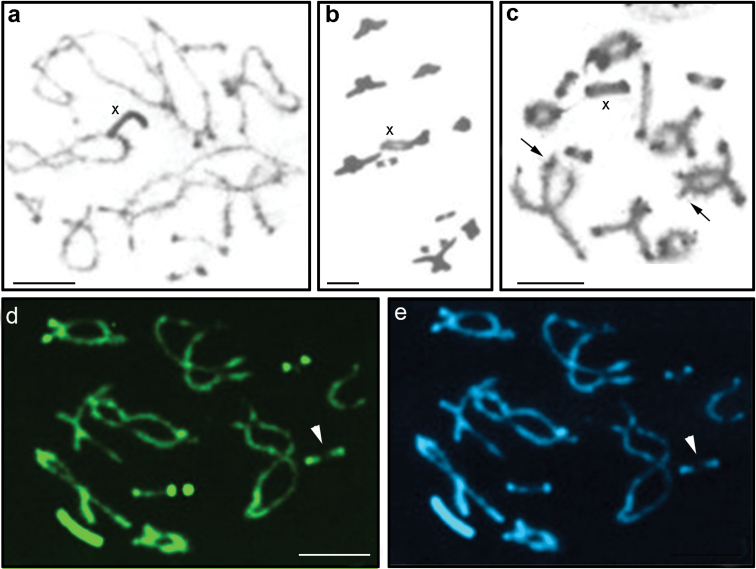
Meiotic stages of *Tropidacris collaris*. **a** pachytene **b** diakinesis **c** C-banding **d** CMA_3_ staining **e** DAPI staining. Arrows indicate C^+^ subterminal blocks in larger pairs and in M_4_. Arrowhead shows the heterozygous form. Bar = 10 µm.

The analysis of mitotic and meiotic chromosomes of *Tropidacris collaris* revealed 2n=24, XX (Fig. 2a) and 2n=23, X0 in females and males respectively. All chromosomes were acrocentric and arranged in three groups according to size: two large (L_1_-L_2_), six medium-sized (M_3_-M_8_) and three small pairs (S_9_-S_11_). The X was the largest among the medium chromosomes. In male meiosis, the X univalent was positively heteropycnotic, as it was observed in diplotene and diakinesis (Fig. 3a, b).

Heterochromatic blocks revealed by C-banding were located in the pericentromeric regions of all chromosomes and M_4_ showed the largest heterochromatic block. The medium-sized chromosomes carried small distal blocks, except for M_8_ without distal blocks, and the X chromosome with a large distal block (Fig. 2b). Additionally, in meiosis, the bivalents L_1_, L_2_ and M_4_ exhibited small interstitial subterminal blocks (Fig. 3c).

The triple staining CMA_3_/DA/DAPI revealed CMA_3_^+^ blocks on the bivalents M_4_, S_10_ and S_11_, the first block being of lower intensity (Fig. 3d). CMA_3_^+^ blocks, which were located in the bivalents S_10_ and S_11_, coincided with the nucleolus organizer regions (NORs). However, a single individual was heterozygous for one of the NOR bivalents (Fig. 3d). DAPI showed homogeneous staining of all chromosomes (Fig. 3e). NORs were restricted to the distal regions of the bivalent S_10_ and pericentromeric region of S_11 _(Fig. 5a), both demonstrating nucleolar activity in all cells analyzed. In addition, Ag-NOR staining revealed a kinetochore marking in S_10 _(Fig. 5a) and another marking (presumably also indicating a nucleolus) at the opposite end of this bivalent.

Fluorescence *in situ* hybridization (FISH) revealed two markings in S_10_ and S_11_ coinciding with the NORs (Fig. 5b). However, the pattern visualized by FISH in the two analyzed samples showed differences in size of the signal involving one of the bivalent homologues, probably S_10_ that carried NORs, indicating a heterozygous condition of the rDNA site on this chromosome (Fig. 5b). FISH with histone H3 probe gene revealed sites on the bivalents M_6_, S_10_ and S_11,_ all located in the proximal position (Fig. 5c).

### Tropidacris cristata grandis

All samples of *Tropidacris cristata grandis* exhibited 2n=23 in males and 2n=24 in females, featuring a sex chromosome system of the X0/XX type (Fig. 2c). Two pairs were of large size (L_1_-L_2_); seven were medium-sized (M_3_-M_9_) and two pairs were small-sized (S_10_-S_11_); all chromosomes were acrocentric. The X chromosome was medium-sized and also acrocentric (Fig. 2c).

**Figure 4.**
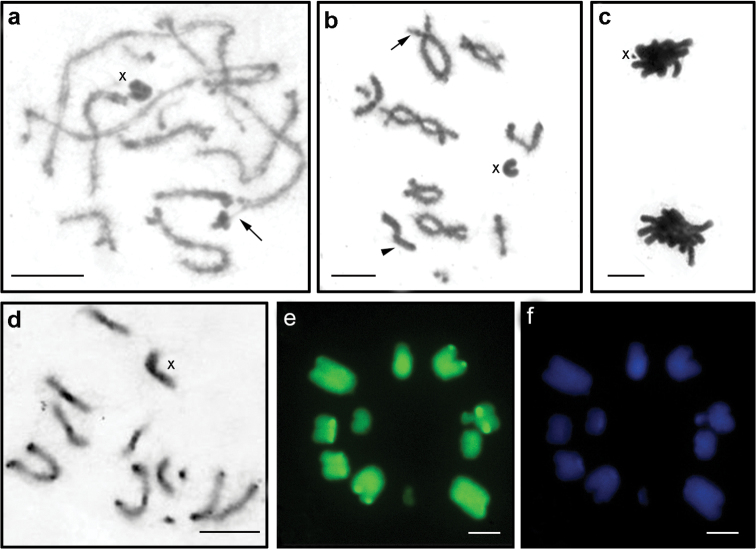
Meiotic stages of *Tropidacris cristata grandis*. **a** pachytene with positively heteropycnotic X chromosome **b** diplotene/diakinesis **c** two daughter cells at anaphase I with visible X chromosome in one of them **d** C-banding with pericentromeric and terminal blocks of heterochromatin in most chromosomes and positively heteropycnotic X chromosome **e** CMA_3_ staining **f** DAPI staining. Arrows and arrowheads indicate secondary constrictions and the megameric chromosome respectively. Bar = 10 µm.

The analysis of meiotic cells in males revealed eleven bivalents corresponding to the autosomes at the pachytene stage; one positively heteropycnotic univalent (the X chromosome); and a structure in a particular bivalent pointing to a secondary constriction (Fig. 4a). At diplotene/diakinesis, eleven bivalents were present. One of them carried a secondary constriction, and the X chromosome remained univalent and positively heteropycnotic (Fig. 4b). Furthermore, one to three chiasmata per bivalent were observed, and one of the autosomes showed a degree of condensation very similar to the X chromosome demonstrating megameric characteristics. In anaphase I, it was possible to observe the correct migration of all autosomes and the presence of the X chromosome in only one of the two resulting cells. This corroborates the occurrence of an X0/XX system (Fig. 4c).

Heterochromatin revealed by C-banding was observed mainly as pericentromeric bands in all chromosomes. In pairs L_1_, L_2_, M_4_, M_8_, S_10_ and S_11,_ these bands were small while they were more developed in the other pairs. Terminal heterochromatic bands were observed on the long arm of pairs L_1_, L_2_ and M_3. _However, a heteromorphic pair was found in two specimens, where one of the L_1_ homologues did not carry this heterochromatic band. Furthermore, two pairs showed discrete bands on the long arm, one distal on pair L_2_ and another (proximal) on the X chromosome (Fig. 2d). In meiocytes, heterochromatic blocks were detected in the pericentromeric and terminal regions, and a more dense heterochromatic region corresponded to the X chromosome (Fig. 4d).

At metaphase II, fluorochrome staining showed terminal GC-rich blocks on L_1_ and L_2_ pairs and on three other chromosomes with CMA_3_^+^ blocks in the proximal regions (Fig. 4e). The AT-rich bands were more discernible than GC-rich bands and were observed only in the pericentromeric regions of five bivalents. In a medium-sized pair, this band was adjacent to a CMA_3_^+ ^region (Fig. 4f). A medium-sized chromosome carried a CMA_3_^+^/DAPI^-^ band, in addition to the terminal CMA_3_^+ ^regions syntenic with the pericentromeric DAPI^+^ bands (Figs 4e and 4f).

**Figure 5.**
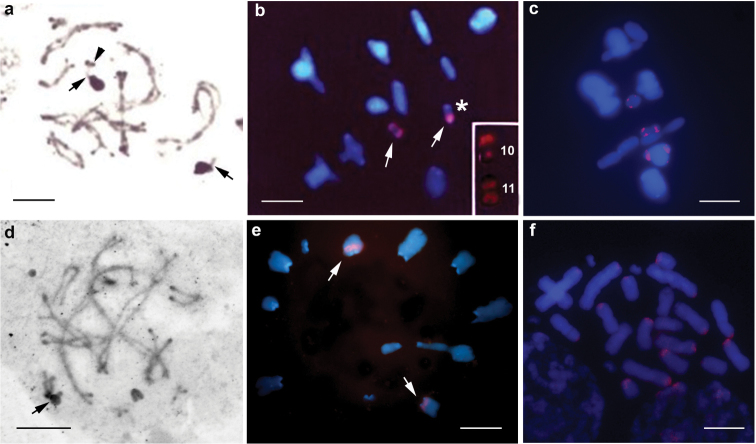
Mitotic and meiotic cells of *Tropidacris collaris* (**a, b, c**) and *Tropidacris cristata grandis* (**d, e, f**). **a, d** silver nitrate impregnation **b, e** FISH with 18S rDNA probe **c, f** FISH with histone H3 gene probe. Black and white arrows, arrowheads and asterisk indicate Ag-NOR bands, rDNA sites, pericentromeric regions and chromosome pair no. 10 respectively (heterozygous condition shown in the box). Bar = 10 µm.

Active NORs were found in one or two bivalents at pachytene (Fig. 5d). FISH with 18S rDNA probe detected rDNA clusters in two bivalents of medium size during metaphase II, M_5_ and M_6_, confirming occurrence of multiple NORs in the heterochromatic regions of this species (Fig. 5e). The histone H3 gene probes hybridized in the pericentromeric regions of most chromosomes, except for the two medium-sized and two small pairs (Fig. 5f).

## Discussion

The observed diploid number (2n♂=23, X0) and the overall structure of the karyotype containing only acrocentric chromosomes were identical in the two species. These karyotypes are similar to those reported for most species of Romaleidae (Mesa et al. 1982, Souza and Kido 1995, Loreto et al. 2005). However, in *Xestotrachelus robustus* (Bruner, 1911), pairs S_9_ and S_10_ are meta- or submetacentric and originated by pericentric inversion. This species, therefore, retains the same chromosome number (Souza et al. 2003).

Although *Tropidacris collaris* and *Tropidacris cristata grandis* have the same chromosome number, they can differ in the karyotype structure. Both species have two pairs of large chromosomes. However, the karyotype of *Tropidacris collaris* contains six pairs of medium-sized chromosomes (M_3_-M_8_) and three small pairs (S_9_-S_11_). On the other hand, *Tropidacris cristata grandis* has seven medium-sized pairs (M_3_-M_9_) and two small pairs (S_10_-S_11_). Furthermore, the conventional analysis of meiocytes revealed two other differences between these species. Specifically, *Tropidacris cristata grandis* has a large chromosome with a secondary constriction, and a megameric chromosome. Grasshoppers of the family Romaleidae have conserved karyotypes, i.e., they reveal extensive uniformity in the chromosome number and chromosomal morphology. However, we found an extensive interspecific variability as regards chromosome banding in the species studied, which indicates a wide variation in the distribution of repetitive DNA sequences. Such variation was also observed in several other members of the group (Vilardi 1988, Souza and Silva-Filha 1993, Souza and Kido 1995, Souza et al. 1998, Pereira and Souza 2000, Souza et al. 2003).

*Tropidacris collaris* and *Tropidacris cristata grandis* have pericentromeric C-bands in all chromosomes. However, we also noted some differences in the distribution of other heterochromatic blocks (Figs 2b and d). Specifically, a pair of chromosomes of *Tropidacris collaris* carried a large pericentromeric block of heterochromatin. On the other hand, similar blocks were detected in five pairs of *Tropidacris cristata grandis* chromosomes, demonstrating that increase in the amount of heterochromatin appears to be more active in the latter species. Moreover, differences in the distribution of other bands were also observed. In *Tropidacris cristata grandis*, the structure of L_1_ chromosome varied among different individuals. For example, one of the homologues did not carry the terminal heterochromatic band. This could be explained in two ways: (i) either by a deletion of this terminal heterochromatic region; or (ii) this chromosome could represent the initial phase of heterochromatinization, where only one of the homologues has the terminal heterochromatic segment. Judging from the heterochromatin distribution in the two other species, these divergences can originate by amplification, multiple replications, unequal crossing-over, accumulation and elimination (John 1988).

Allopatric speciation usually occurs after the relatively long geographical isolation between populations. Consequently, these populations accumulate genetic differences that can cause reproductive incompatibilities (Presgraves 2010). These genetic differences may be related either to gene mutations or to changes in the chromosome constitution, such as heterochromatin spreading in both autosomes and sex chromosomes (Presgraves 2010, Molina et al. 2012, Pucci et al. 2014). Schweizer and Loidl (1987) proposed that heterochromatin transfer between equilocal regions might occur between non-homologous chromosomes of similar size due to the positioning of these chromosomes in the nucleus bouquet configuration. The transfer of the pericentromeric heterochromatin probably happened in *Tropidacris* species studied in this paper. The expansion of heterochromatin could also occur due to transposable elements, which are genetic determinants of heterochromatin formation in different organisms (Grewal and Jia 2007). Then, geographical isolation occurred in the ancestral populations of this genus, as proposed by Carbonell (1986). This, in turn, enabled heterochomatinization that led to differences in the chromosome constitution observed in the two species. The action of geological forces added to the current fragmentation of the environment and, therefore, allowed the formation of allopatric populations, promoting speciation in the genus.

Other differences were found between the two species with respect to the base content of DNA that constitutes heterochromatin. CMA_3_^+^ blocks (GC-rich) were observed in three chromosomes of *Tropidacris collaris*, while *Tropidacris cristata grandis* showed a higher number of bands. These results indicate that, in addition to the expansion of the heterochromatin, its composition has also been modified in terms of base pairs. The occurrence of DAPI^+^ heterochromatin (AT-rich) in *Tropidacris cristata grandis* also differentiates it from *Tropidacris collaris*, since the karyotype of the latter species does not have AT-rich regions. The same is true for the various members of this family studied to date (Loreto et al. 2008, Rocha et al. 2011, Bueno et al. 2013). Likewise, the DAPI^+^ blocks of the chromosomes M_3_ and M_5_ of *Tropidacris cristata grandis* were syntenic with CMA_3_^+^ blocks. These results indicate the presence of at least three different compositions of heterochromatin in this species; GC-rich, AT-rich, and that with interspersed AT and GC-rich segments. In *Tropidacris collaris*, there are two types of heterochromatin composition: a GC-rich one and another neutral for AT, e.g., all chromosomes with homogeneous staining. These results reinforce the differences between the two species, where the pattern found in *Tropidacris collaris* is more similar to that observed in most species of Romaleidae (Souza et al. 1998, Pereira and Souza 2000, Souza et al. 2003, Anjos et al. 2013). *Tropidacris collaris* has a wider distribution, and in *Tropidacris cristata grandis* it is geographically more restricted. Thus, gene flow is higher in *Tropidacris collaris* and, therefore, chromosomal rearrangements are not easily established within different populations of this species.

Most studies of histone H3 genes in grasshoppers reveal a localization of these sequences on a single pair of chromosomes (Cabrero et al. 2009, Cabral-de-Mello et al. 2011a, 2011b, Regueira-Neto et al. 2013). In *Tropidacris collaris* and *Tropidacris cristata grandis*, these genes were observed in more than one pair, and histone H3 genes were always found in terminal regions of 14 autosomes and the X chromosome in the latter species. A similar situation was reported for the grasshoppers *Abracris flavolineata* (De Geer, 1773) (Bueno et al. 2013) and *Rhammatocerus brasiliensis* (Bruner, 1904) (Oliveira et al. 2011). The difference in the distribution of histone genes leads to the assertion of a possible amplification of this gene family. Moreover, the distribution of the histone genes corresponds to heterochromatin location and may show an intercalary distribution of these sequences. This heterochromatic region consists of highly condensed chromatin, where few mRNAs are produced and there is abundance of repetitive elements, such as satellite DNA and transposons (Pikaard and Pontes 2007). The association between genes and repetitive sequences, such as histone H3 genes, could result in variation in the number of copies of these genes. Thus, an increase in unequal exchanges and gene duplication might occur (Zoldos et al. 1999, Hamon et al. 2009). Moreover, the transposable elements of the heterochromatic regions could contribute to the dispersion of histone genes to several chromosomes, as observed in different genetic sequences (Sczepanski et al. 2013, Barbosa et al. 2015).

The wide range of variation in heterochromatin among the samples of the two species, in addition to the distribution of GC-rich blocks and co-localization with H3 histone genes in *Tropidacris cristata grandis*, is an exceptional feature in grasshoppers. Heterochromatin has the ability to spread to different regions and influence gene expression, leading to silencing of some genes and preventing recombination between them (Gottlieb and Esposito 1989, Grewal and Jia 2007). The presence of multiple copies of histone H3 genes in the heterochromatic regions may indicate that these copies are silenced. The co-localization with CMA_3_^+^ regions is indicative of silencing, since DNA methylation occurs preferentially in GC-rich regions and also influences gene silencing (Newell-Price et al. 2000, Schotta et al. 2002), thus providing a balance in the processes of expression of the histone H3 genes.

The NORs were observed in two chromosome pairs in both species, although these pairs are different. While *Tropidacris collaris* has NORs on two small pairs, these structures were observed on medium-sized chromosomes of *Tropidacris cristata grandis*. Regueira-Neto et al. (2013) suggested that the location of NORs on medium-sized autosome pairs is the ancestral location of the 45S rDNA genes in Romaleidae. Thus, location of rDNA sites on small chromosome pairs of *Tropidacris collaris*, as well as the presence of multiple copies on more than one autosomal pair indicate the occurred rearrangements and amplification of the sequences associated with the spread of heterochromatic regions.

The data presented in this study demonstrate karyotype conservation regarding the chromosome number and morphology in both species of *Tropidacris* when compared to other species of Romaleidae. However, they indicate that *Tropidacris cristata grandis* has an extremely diverse karyotype in terms of the presence and distribution of heterochromatic blocks and differentiation in the localization of histone H3 genes, showing karyotypic differences from *Tropidacris collaris*. While *Tropidacris collaris* is widely distributed in Brazil, *Tropidacris cristata grandis* has a restricted geographical distribution within isolated fragments of the rainforest. Originally, the Atlantic Forest extended along almost the whole east coast of Brazil, with extensive incursions into the inner parts of the country (Morellato and Haddad 2000). Nowadays, it is reduced to a set of small remaining fragments, usually less than 100 hectares, which are isolated and subject to intense edge effect (Ribeiro et al. 2009). The restricted distribution of this species is associated with the devastation of these environments and can lead to geographical isolation of the resulting populations. This isolation can promote establishment of the above-mentioned karyotypic changes and launch the speciation process in *Tropidacris cristata grandis*, which becomes increasingly divergent from the other species of the genus.
